# Introduction to Nanoscale Advances in Innovative Bioengineering

**DOI:** 10.1039/d6na90017h

**Published:** 2026-03-04

**Authors:** Zhen Gu, Zhicheng Le, Zheng Su

**Affiliations:** a School of Chemistry and Biological Engineering, Beijing Key Laboratory for Bioengineering and Sensing Technology, University of Science and Technology Beijing Beijing 100083 P. R. China; b School of Biomedical Engineering, Shenzhen Campus of Sun Yat-sen University Shenzhen Guangdong 518107 P. R. China; c The First Affiliated Hospital of USTC, Division of Life Sciences and Medicine, University of Science and Technology of China Hefei Anhui 230001 P. R. China

## Abstract

Zheng Su, Zhicheng Le and Zhen Gu introduce the *Nanoscale Advances* themed issue on Nanoscale Advances in Innovative Bioengineering.
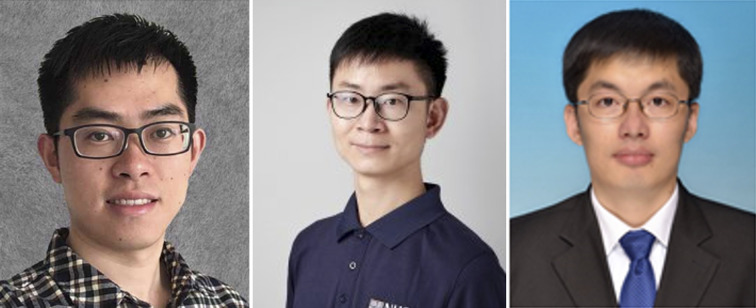

Over the past few years, “innovative bioengineering” has entered a phase of rapid acceleration because we can increasingly engineer biological function at the same length scales where key decisions are made, including for molecular transport, adhesion, mechanotransduction, electrical/ionic signalling, and immune recognition. In parallel, nanoscale materials, together with micro/nano-fabrication, are transforming biological interfaces from passive contact regions into tunable, information-rich control layers that support bidirectional exchange between devices/materials and living systems. This shift is also changing what “translation” looks like: beyond improving biocompatibility, the field is moving toward manufacturable, testable, and standardizable platforms that can be integrated into real workflows, scaled with quality control, and evaluated using meaningful functional endpoints. Viewed together, the papers in this collection can be connected through a closed-loop perspective in which nanoscale materials and structures enable controllable fabrication, which in turn defines programmable biointerfaces and ultimately drives bioengineering impact in realistic settings.

A first thematic cluster highlights the evolution from materials that merely remain stable in physiological environments to interfaces that actively communicate with biology. Across diverse modalities (electrical, mechanical, chemical, and hybrid), nanoengineering provides a shared toolkit: (i) tailoring surface chemistry and nanoscale topography to guide protein adsorption and cellular responses; (ii) embedding functional nano-domains that transduce energy or signals across the interface; and (iii) integrating these capabilities into miniaturized architectures compatible with real-world constraints, such as implantation routes, operating time, and power budgets. Importantly, the core advance is not only higher performance in controlled experiments, but also the emergence of translational design logic-interfaces that are increasingly judged by whether they can be benchmarked quantitatively, reproduced reliably, and integrated into clinically relevant procedures or wearable workflows. In this sense, nano-biointerface design is becoming a systems problem in which nanoscale choices propagate to manufacturability, reliability, and end-user adoption. Wireless, field-controlled biointerventions are particularly attractive for constrained clinical settings such as interventional cardiology. In this collection, Suarato and co-workers investigate the feasibility of magnetoelectric nanoparticles as magnetically mediated nanoscale electroporation and drug-release agents using computational analysis of bioelectric phenomena at the nanoparticle–tissue interface (https://doi.org/10.1039/D5NA00438A).

Building on nanoengineered interfaces, a second cluster emphasizes nanoscale-enabled biofabrication and 3D living models, where nanoscale components act as programmable building blocks rather than passive additives. In nanobioprinting and engineered tissue systems, the central challenge extends beyond geometric fidelity to the recreation of microenvironments that control phenotype over time, such as for mechanics, diffusion, cell–matrix signalling, and dynamic remodelling. Nanomaterials can expand the design space of bioinks and scaffolds by enabling multi-functionality, localized cues, and embedded readouts, while advances in manufacturing translate these designs into reproducible constructs. A pragmatic consideration also emerges: for complex living models to become broadly useful in bioengineering and translational research, they must be robust, comparable across laboratories, and distributable. Consequently, standardizable workflows for culture, characterization, storage, and transport are becoming as important as the initial fabrication itself, and nanoscale strategies are well positioned to unlock improvements in reliability and accessibility. To address the practical bottleneck of scaling complex living models, He, Sun and co-workers survey how nanomaterials can support both organoid culture and cryopreservation, while outlining remaining challenges in biocompatibility, scalability, long-term safety, and standardization (https://doi.org/10.1039/D5NA00534E).

Complementary to these interface and manufacturing advances, a third thread connects nanoscale design to cellular reprogramming and immuno/gene engineering, where controllable delivery remains a persistent barrier. Here, nanoscale parameters—including size, degradability, ligand presentation, and charge distribution—are leveraged to navigate uptake pathways, intracellular trafficking, and functional outcomes while preserving cell health. The bioengineering value of this direction lies in its emphasis on tunability and workflow integration: delivery systems are increasingly evaluated as modular platforms that can be adapted across payloads and cell types, and that can be embedded within reproducible processing pipelines and quality-control frameworks. This platform mindset naturally connects to translation, because immune and gene-engineered products demand stringent manufacturing reproducibility and safety profiling. Extending this platform mindset into cell and gene engineering, Gharatape and co-workers synthesize and evaluate low-molecular-weight poly(β-amino ester) nanocarriers and assess their potential for enhanced T-cell transfection and gene delivery in the context of cancer immunotherapy (https://doi.org/10.1039/D5NA00169B).

Looking forward, the field’s largest gains will come from converting elegant nanoscale concepts into repeatable, manufacturable, and clinically navigable platforms. Key opportunities and challenges include: standardization of quantitative metrics (*e.g.*, biointerface performance, printing fidelity, and potency/viability endpoints) to enable cross-lab comparability; manufacturability and quality control from scalable synthesis to batch-to-batch validation; long-term safety, including biocompatibility, immune interactions, and, when applicable, predictable biodegradation and clearance; clearer regulatory and clinical pathways grounded in realistic preclinical models and fit-for-purpose endpoints; and deeper cross-disciplinary collaboration in which materials scientists, engineers, and clinicians co-design around workflow constraints from the outset.

As Guest Editors, we thank all authors for their excellent contributions and the reviewers for their careful evaluations. We are also grateful to the *Nanoscale Advances* editorial team for their consistent guidance and support in assembling and promoting this collection.

